# Intrauterine lidocaine and naproxen for analgesia during intrauterine device insertion: randomized controlled trial

**DOI:** 10.1186/s40834-019-0094-0

**Published:** 2019-09-10

**Authors:** Shana M. Miles, Katerina Shvartsman, Susan Dunlow

**Affiliations:** 1 Second Medical Group, 243 Curtiss Rd, Barksdale AFB, Louisiana, 71110 USA; 2 Department of Obstetrics and Gynecology, Uniformed Services University, 4301 Jones Bridge Rd, Bethesda, MD 20814 USA; 3 Department of Obstetrics and Gynecology, Walter Reed National Military Medical Center, 8901 Wisconsin Ave, Bethesda, MD 20889 USA

**Keywords:** Intrauterine device, IUD, Pain, Lidocaine, NSAID

## Abstract

**Background:**

This study evaluates oral naproxen and intrauterine instillation of lidocaine for analgesia with intrauterine device (IUD) placement as compared to placebo.

**Methods:**

This was a randomized, double-blind, placebo-controlled trial. Patients desiring levonorgestrel 52 mg IUD or Copper T380A IUD were randomized into treatment groups. Patients received either oral naproxen 375 mg or placebo approximately 1 h prior to procedure in conjunction with 5 mL of 2% lidocaine or 5 mL of intrauterine saline. The primary outcome was pain with IUD insertion measured on a visual analog scale immediately following the procedure. Prespecified secondary outcomes included physician pain assessment, post procedure analgesia, satisfaction with procedure, satisfaction with IUD, and pain assessment related to IUD type.

**Results:**

From June 4, 2014 to October 28, 2016 a total of 160 women desiring Copper T380A or levonorgestrel 52 mg intrauterine device insertion and meeting study criteria were enrolled and randomized in the study. Of these, 157 (78 in the Copper T380A arm, 79 in the levonorgestrel 52 mg) received study treatment medication. There were 39 in naproxen/lidocaine arm, 39 in placebo/lidocaine arm, 40 in naproxen/placebo arm, and 39 in placebo/placebo arm. There were no differences in the mean pain scores for IUD placement between treatment groups (naproxen/lidocaine 3.38 ± 2.49; lidocaine only 2.87 ± 2.13; naproxen only 3.09 ± 2.18; placebo 3.62 ± 2.45). There was no difference in self-medication post procedure or in satisfaction with the procedure and IUD among women in the treatment arms or by type of IUD.

**Conclusion:**

Naproxen with or without intrauterine lidocaine does not reduce pain with IUD placement.

**Clinical trial registration:**

Clinicaltrials.gov, NCT02769247. Registered May 11, 2016, Retrospectively registered

## Background

Approximately 50% of pregnancies in the United States are unintended [1, 2] with over 4 million women at risk of unintended pregnancy not utilizing contraception [3]. Intrauterine devices (IUDs) are among the most effective long-acting reversible contraceptives [4]. Currently, five IUDs are available in the United States: the Copper T380A (Paragard), levonorgestrel 52 mg (Mirena), levonorgestrel 52 mg (Liletta), levonorgestrel 19.5 mg (Kyleena) and levonorgestrel 13.5 mg (Skyla). The safety, efficacy, convenience, and cost of long acting reversible contraception, including IUDs, has led the Institute of Medicine to include contraception as part of women’s preventive care in the Affordable Care Act in an effort to decrease barriers to care [5].

While IUD and contraceptive implant use has grown over the past decade, the current usage is only 14% in the United States [6]. One of the barriers to IUD insertion is discomfort, or anticipation of pain, during device insertion [7–9]. While most patients tolerate pain to complete the procedure, studies show that pain scales on IUD insertion range from 2 to 7 on a 10 point visual analog score [10–15]. Surprisingly, adequate pain control during gynecologic outpatient office procedures including hysterosalpingography, endometrial biopsy, and IUD insertion and removal have been addressed in only a limited way in the literature. Moreover, despite the discomfort associated with IUD insertion, endometrial biopsy, and other office gynecology procedures, there is no standard of care for pre/intra/post procedure analgesia. In our institution, depending on provider preference and previous patient counseling, many patients receive no pain medication for their IUD insertion procedure.

Autonomic innervation to the uterus and cervix is provided via S2-S4, and the T10-L1 afferent visceral pain fibers [16]. Intrauterine instilled anesthetic is thought to act on nerve endings within the endometrium [17]. As such, analgesia for common office outpatient gynecologic procedures that have been investigated include: intracervical or paracervical injections, nonsteroidal anti-inflammatory drugs (NSAIDs), intrauterine instillation. Despite efficacy with intracervical/paracervical injections [18], the injection is painful and is a deterrent to use [19–21]. NSAIDs block prostaglandin synthesis and have been shown to be effective in relief of mild-moderate pain associated with gynecologic procedures [20]. A 2015 Cochrane review examining analgesia with IUD insertion found that naproxen may have an effect at reducing pain during IUD insertion [22] however, subsequently, Ngo et al. found that while naproxen did not reduce pain with IUD insertion it did reduce pain post-insertion [13].

Intrauterine instillation of lidocaine has been shown to be efficacious in endometrial biopsy [23–25], saline sonogram [26], and retrieval of “lost” IUD [27]. Another study showed that intrauterine lidocaine in conjunction with naproxen significantly decreased the pain associated with endometrial biopsy [25]. If the administration of oral and/or intrauterine analgesia was efficacious for intrauterine device insertion, this would be a low cost and a low risk intervention. The aim of this study was to determine efficacy of NSAID and intrauterine analgesia, either singly or doubly, during intrauterine device insertion on patients’ pain perception.

## Materials and methods

This study was approved by the Walter Reed National Military Medical Center Institutional Review Board (#384645–3) prior to enrollment of the first patient and the study was posted on Clinicaltrials.gov (NCT02769247) on May 11, 2016. This was a factorial design, randomized, double blind, placebo-controlled study with women who were recruited from our outpatient obstetrics and gynecology clinics. Prior to their enrollment in the study for the intrauterine device of their choice (Copper T380A or Mirena levonorgestrel 52 mg), subjects signed procedure consent. After subjects completed a study consent form with a study physician in accordance with the Declaration of Helsinki, they completed a demographics form to ensure that they met inclusion criteria and had no exclusion criteria. Inclusion criteria included Defense Enrollment Eligibility Reporting System (DEERS)-eligible women aged 18 years and older desiring Copper T380A or levonorgestrel 52 mg intrauterine device insertion. DEERS eligibility would allow them to receive care at a Military Treatment Facility. Exclusion criteria were current pregnancy, history of cervical stenosis, severe medical illness, known allergy or sensitivity to lidocaine or naproxen, peptic ulcer disease, current pelvic inflammatory disease, patients with known renal insufficiency, and patients using chronic NSAIDs or on chronic pain medication. Due to the infrequency of Skyla IUD use in our clinic and the non-availability of Liletta and Kyleena at the time of the study, women desiring these devices were also excluded.

Each subject was then assigned a study number and was randomized to a treatment within the Copper T380A or Levonorgestrel 52 mg arms by the investigational pharmacy on the day of enrollment. The patient chose which IUD they desired prior to enrollment and was not randomized to IUD type. The investigational pharmacist, who was otherwise not involved in the study, used computer generated block randomization. The pharmacy provided study packets that included an unlabeled syringe and oral medication so that physicians and patients were blinded to the assigned treatment group. The subject took the oral medication (naproxen 375 mg prepared in a capsule or similarly prepared placebo capsule) 1 h prior to the procedure. After performing a bimanual exam, placing a speculum and cleansing the cervix with betadine, the study physician then instilled 5 ml of the intrauterine solution (2% lidocaine or similarly prepared normal saline) through the endocervix using an 18-gauge angiocatheter advanced to the hub. The angiocatheter was left in place for 3 min before it was removed. Single tooth tenaculum was then placed and the uterus was measured with a metal sound. The intrauterine device was then inserted according to manufacturer’s instruction. To maintain consistency, three study physicians (KS, SD, SM) used the same technique to place the IUDs.

Immediately after the procedure, each patient completed a post procedure survey rating their pain during the procedure, satisfaction with the insertion, and whether they would have an IUD placed again. The survey included a visual analog scale (VAS) where 0 represented no pain and 10 being the worst pain they could imagine. The satisfaction ratings were scored on a scale from 1 to 5 with 5 being extremely satisfied with the procedure. The patients completed another survey at day 30 post insertion. The physician also recorded and scored visible signs of the women’s discomfort during the procedure (gripping table, lifting off table) using a 3-point observer scale for each of the parameters [24]. On this scale 0 was no response, 1 was patient admits to some discomfort but procedure is not interrupted, 2 was patient in discomfort but after briefly halting the procedure, the IUD is inserted while 3 the patient is visibly uncomfortable, gripping the table or squeezing the attendant’s hand. The difficulty of insertion was also noted. Lidocaine and saline were packaged in identical syringes and oral placebo tablets were encased in same capsule as the naproxen. Patients and physicians were blinded to treatment groups but not IUD type.

Power calculation was based on other studies assessing pain with IUD placement and endometrial biopsy [11, 21, 25]. Power calculation was done for treatment groups of 34 patients, assuming pain rating in placebo group of 4, standard deviation of VAS scores is 2.9 points, with a two-sample t test with 5% two-sided significance level will have 80% power to detect a difference of 2 points between the groups. Clinically significant difference in VAS pain score has been defined as 2 on a 0–10 VAS scale [28, 29]. To allow room for missing data, non-completion of procedure and loss to follow up, we planned to recruit 80 women in each IUD type (levonorgestrel 52 mg vs Copper T380A) and 40 women in each treatment arm (placebo/normal saline; naproxen/normal saline, placebo/lidocaine; naproxen/lidocaine), for a total of 160 patients.

Demographics and survey data were compared using the t test or Wilcoxon two-sample test for continuous variables. X^2^ or Fisher exact test was used for categorical variables. Additional statistical analysis was performed using Kruskal-Wallis test to evaluate the differences of the mean pain scores between the groups. *P* < 0.05 was considered statistically significant.

## Results

**Fig. 1:**
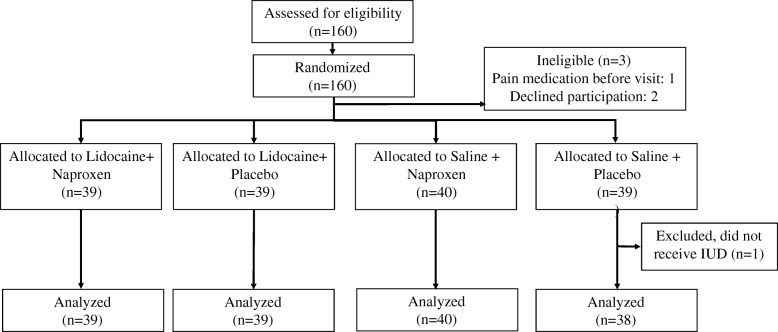
Patient enrollment and randomization to groups. Flow diagram, per Consolidated Standards of Reporting Trials. IUD, intrauterine device

There were 160 women enrolled in the study between June 2014 and October 2016, with 80 desiring the levonorgestrel 52 mg IUD and 80 desiring Copper T380A IUD with 40 randomized in each treatment arm. Patients chose their desired IUD prior to enrollment. Enrollment was closed for either levonorgestrel 52 mg IUD or Copper T380A IUD after 80 women were enrolled in each respective arm. Two patients decided against study participation after enrollment, another took non-study medication prior to her IUD insertion excluding her from the analysis, and another was unable to have her IUD placed due to difficulty with IUD insertion (Fig. 1). All other patients analyzed received the assigned study medication and completed IUD insertion. A total of 157 patients were analyzed (79 patients desiring levonorgestrel 52 mg IUD, 78 patients desiring Copper T380A IUD). Within treatment groups there were 39 in the combined treatment arm, 39 in the intrauterine lidocaine only arm, 40 in the naproxen only arm, and 39 in the placebo arm. There were no statistically significant differences among treatment groups in age, body mass index (BMI), parity, history of cervical procedures, vaginal parity, ease of insertion, use of pain medication post-procedure as shown in Tables 1 and 2. There was a significant difference of distribution of patients with history of c-section (*p* = 0.031) between treatment groups.

**Table 1:** Patient demographics

Characteristic	Lidocaine/Naproxen (*n* = 39)	Lidocaine/Placebo (*n* = 39)	Saline/Naproxen (*n* = 40)	Saline/Placebo (*n* = 39)	P
Age (y)	31.1 ± 8.5	31.1 ± 7.4	31.2 ± 6.5	29.7 ± 6.9	0.788
BMI (kg/m^2^)	27.6 ± 6.4	26.0 ± 4.1	27.1 ± 5.1	25.9 ± 4.4	0.340
Parity					0.510
Nulliparous	13 (33.3)	17 (43.6)	11 (27.5)	14 (35.9)	
Parous	26 (66.7)	22 (56.4)	29 (72.5)	25 (64.1)	
Vaginal parity	18 (46.1)	15 (38.4)	12 (30.0)	14 (35.9)	0.408
Prior c-section	10 (25.6)	9 (23.1)	20 (50.0)	14 (35.9)	0.031
Prior IUD insertion					0.605
Yes	6 (15.4)	9 (23.0)	12 (30.0)	8 (20.5)	
No	33 (84.6)	30 (76.9)	28 (70.0)	31 (79.5)	
Prior Cervical procedures					0.298
Yes	5 (12.5)	12 (30)	6 (15)	6 (15.4)	
No	34 (85)	27 (67.5)	34 (85)	33 (84.6)	
Ease of insertion					0.99
Easy	38 (97.4)	38 (97.4)	38 (95)	36 (94.7)	
Difficult	1 (2.6)	1 (2.6)	2 (0.5)	2 (5.2)	

*Data are* n%, unless otherwise specified

*BMI* body mass index, *IUD* intrauterine device

**Table 2:** Analgesic outcomes

Characteristic	Lidocaine/Naproxen	Lidocaine/Placebo	Saline/Naproxen	Saline/Placebo	P
Physician pain assessment	0.77 ± 0.11	0.56 ± 0.09	0.48 ± 0.11	0.59 ± 0.8	0.238
Visual analog pain score	3.38 ± 2.49	2.87 ± 2.13	3.09 ± 2.18	3.62 ± 2.45	0.456
Post procedure analgesia					0.568
Yes	10 (25)	8 (20)	9 (22.5)	12 (30.8)	
No	28 (70)	28 (70)	31 (77.5)	22 (56.4)	
Satisfaction with procedure	4.36 ± 0.84	4.26 ± 0.94	4.3 ± 0.94	4.4 ± 0.9	0.910
Satisfaction with IUD (Day 30)					0.701
Levonorgestrel 52 mg	4.40 ± 0.75	4.36 ± 0.97	4.35 ± 1.1	4.38 ± 0.89	
Copper T380A	4.31 ± 0.95	4.16 ± 0.92	4.3 ± 0.81	4.47 ± 0.94	
Physician pain assessment					0.31
Levonorgestrel 52 mg	0.90 ± 0.72	0.50 ± 0.11	0.55 ± 0.18	0.53 ± 0.12	
Copper T380A	0.63 ± 0.13	0.63 ± 0.14	0.40 ± 0.11	0.63 ± 0.11	
Visual analog pain score					
Levonorgestrel 52 mg	3.55 ± 0.55	2.45 ± 0.44	2.85 ± 0.45	3.26 ± 0.54	0.09
Copper T380A	3.26 ± 0.59	3.32 ± 0.52	3.35 ± 0.52	4.00 ± 0.58	
Post procedure analgesia					
Levonorgestrel 52 mg					0.65
Yes	3 (16) [0.33]	3 (17) [0.23]	5 (25) [0.09]	5 (31) [0.64]	
No	16 (84) [0.09]	15 (83) [0.06]	15 (75) [0.02]	11 (69) [0.18]	
Copper T380A					0.43
Yes	7 (41) [0.45]	5 (28) [0.10]	4 (20) [0.89]	7 (41) [0.45]	
No	10 (59) [0.21]	13 (72) [0.05]	16 (80) [0.42]	10 (59) [0.21]	

*Data are mean* ± standard deviation, n%, [X^2^] unless otherwise specified

**Fig. 2:**
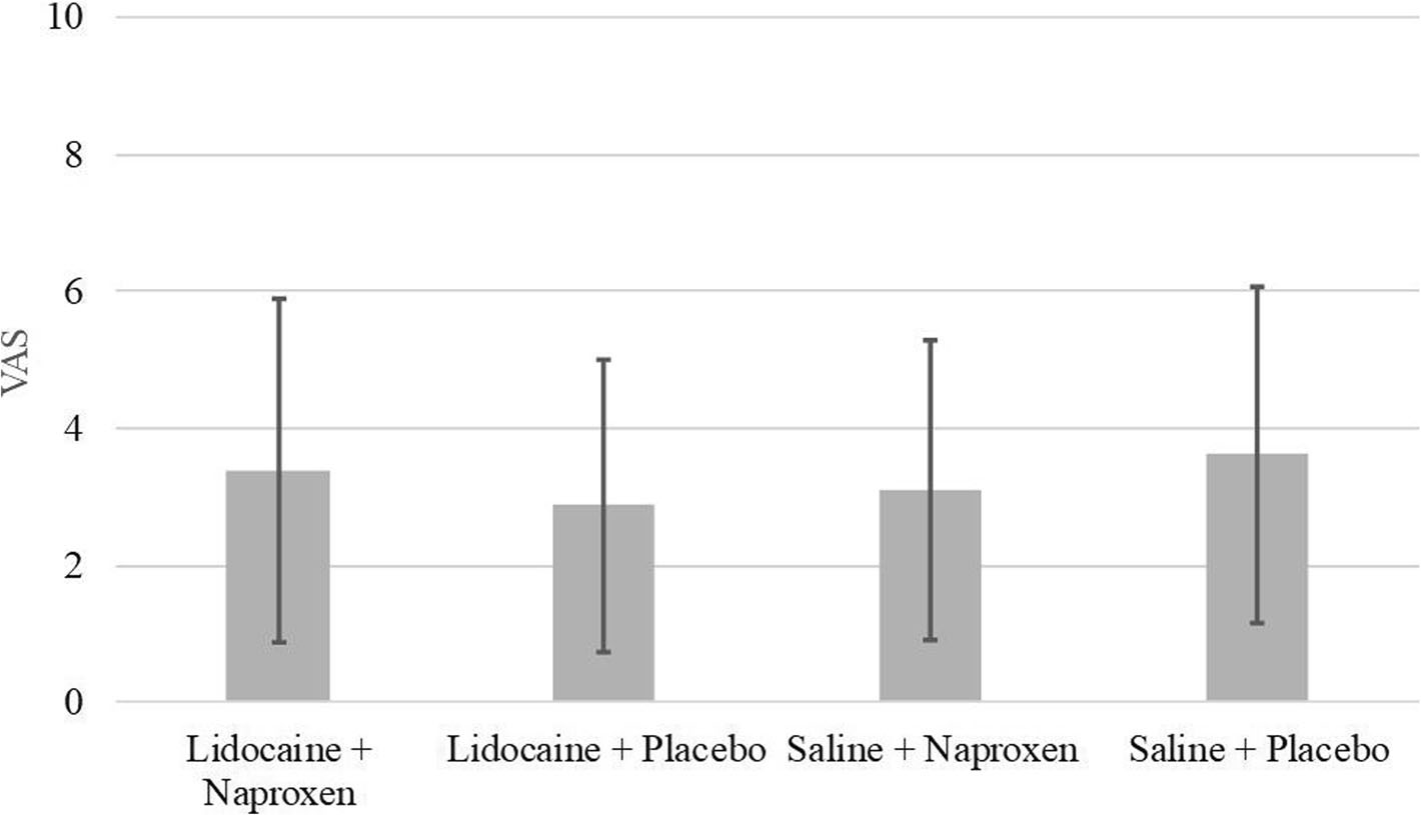
Mean visual analog scores within 30 min of IUD insertion. Bars denote treatment groups with standard deviation. Treatment groups are lidocaine instillation and naproxen, lidocaine instillation and placebo capsule, placebo instillation and naproxen, placebo instillation and placebo capsule

Pain perceived by patients in the treatment groups was not statistically significant between the groups with mean VAS in the placebo arm of *3.62* ± 2.45 (Fig. 2) as compared to the naproxen only (3.09 ± 2.18), lidocaine only (2.87 ± 2.13), naproxen and lidocaine (3.38 ± 2.49) arms. Pain assessment during the procedure rated by the physician inserting the device was not significantly different between treatment groups or type of IUD (Table 2). There was no statistically significant difference in patients’ use of post procedure analgesia regardless of IUD type (*p* = 0.19) or treatment groups (*p* = 0.568) when surveyed approximately 30 days post-insertion. This study was not powered to detect differences between treatment groups between types of IUDs nor were patients randomized to type of IUD.

Overall satisfaction for the IUD procedure was high with 98% of the patients scoring they were satisfied to extremely satisfied with their procedure and 97% would have an IUD placed again. Satisfaction scores were not different between treatment groups or IUD type. There was one patient that was diagnosed with pelvic inflammatory disease approximately 1 week after her IUD insertion and her IUD was inadvertently removed during her requested clipping of strings. Patient’s IUD was not replaced at that visit. Another patient returned with vasovagal symptoms approximately 1 h following placement of her IUD however this did not affect her retention of the device. This study was not powered to detect differences in the low rates of adverse events or measures of patient satisfaction.

## Discussion

Consistent with other studies, the data suggests that there is no significant difference in pain control during IUD insertion using NSAIDs like naproxen [11, 13, 30–32]. The average VAS of 3.6 in our placebo group is consistent with other studies [30, 31]. The wait time of 1 h after administration of naproxen and three minutes with intrauterine instillation should have been appropriate to achieve peak levels of analgesia at time of IUD insertion [33]. To our knowledge, this is the first randomized, double-blind, placebo-controlled study comparing the use of NSAIDs and intrauterine instillation of anesthetic for IUD insertion. There was no observed multiplicative effect as had been previously shown with the same regimen for endometrial biopsy [25].

There was no difference in post-procedure analgesia usage in our treatment groups or between IUD type (Table 2). However, only 26% of our patients used any analgesia post procedure (X^2^ = 0.568) and there were no differences between patients who received naproxen versus placebo. This suggests that naproxen did not have an effect on post procedure analgesia despite its up to 12 h duration of analgesia [33]. However, recall bias may have contributed to this finding since the patients were asked if they took anything additional for pain after their IUD insertion during their 30-day post-procedural survey. There were also no differences in pain ratings or post procedure analgesia in patients receiving the intrauterine lidocaine as compared to saline. This suggests that the lidocaine also does not have a significant effect on analgesia for IUD insertion either during or post-procedure. Interestingly, there was a trend towards decreased usage of post-procedure analgesics in the lidocaine instillation arms within the levonorgestrel IUD treatment groups, however this study was not adequately powered for this outcome measure (Table 2).

Limitations of this study include the fact the placebo group still received instillation of saline which itself may have been a treatment modality. Given the pain ratings of our patients at the lower end of the reported range, this may suggest that the instillation and distension of the uterus may affect the nerve endings within the endometrium and, consequently, patients’ perceived pain rating. The hydro-distention accomplished by the intrauterine instillation may have affected the dilation of the insertion path, thereby making the insertion of the IUD devices easier. This distension may also contribute to the finding of no differences in pain perception between devices. Usage of the 18-gauge angiocatheter was a separate procedure and possible treatment modality. While not specifically tracked there was a small proportion of patients that noted cramping during the instillation though none required the procedure to be paused or halted during this step. Including a separate arm with no intervention other than IUD insertion would have improved this study and addressed this potential confounding factor of intrauterine instillation.

While we had no differences in distribution of parous women throughout our treatment groups, it is also possible that if subjects had been limited to nulliparous women, differences in pain perception may have been noted between the treatment groups in a similarly powered study. This study was not randomized to type of IUD or powered to detect differences between the two devices but we did observe a trend of increased pain (*p* = 0.09) in the Copper T380A patients as compared to the levonorgestrel recipients in conjunction with decreased pain in our naproxen and lidocaine treatment groups. Our findings were similar to Dogan et al. [25], in that the combined treatment group was not superior to single agent. However, unlike the findings from Dogan et al., our naproxen/lidocaine arm was not superior to the placebo/saline arm which maybe secondary to our overall lower pain scores during IUD insertion compared to endometrial biopsy in their study. This study was also similar to the 2015 Cochrane review that found no benefit to pre-treatment with naproxen (NSAID) for analgesia for IUD insertions though patients in the Cochrane review were less likely to note the insertion experience as unpleasant [22].

In conclusion, our study showed that naproxen alone or in combination with intrauterine lidocaine does not reduce pain during IUD insertion. Intrauterine instillation of lidocaine is not superior to saline. A future study examining the difference of intrauterine instillation of saline and no instillation may help elucidate these differences.

## Data Availability

Datasets can be found on Clinicaltrials.gov, http://clinicaltrials.gov, NCT02769247. Any data sets used or analyzed that may not be found on the site are available from the corresponding author on reasonable request.

## Abbreviations

BMI: Body mass index
DEERS: Defense Enrollment Eligibility Reporting System
IUD: Intrauterine device
NSAID: Nonsteroidal anti-inflammatory drug
VAS: Visual analog scale
